# Predictors of Healthcare Workers’ Compassionate Care Amid the COVID-19 Pandemic: A Cross-Sectional Study from Patients’ Perspective in Kelantan, Malaysia

**DOI:** 10.3390/ijerph20021380

**Published:** 2023-01-12

**Authors:** Noorhidayu Monyati Mohamed Noor, Mohd Ismail Ibrahim, Suhaily Mohd Hairon, Maizun Mohd Zain, Mohd Saiful Nazri Satiman

**Affiliations:** 1 Department of Community Medicine, School of Medical Sciences, Universiti Sains Malaysia, Kubang Kerian 16150, Malaysia; 2 Public Health Unit, Hospital Raja Perempuan Zainab II, Kota Bharu 16150, Malaysia; 3 Medical Division, Kelantan State Health Department, Kota Bharu 16150, Malaysia

**Keywords:** compassionate care, relational aspect of care questionnaire Malay version, predictors, COVID-19 pandemic, inpatients, public hospitals

## Abstract

Background: Compassionate care served by healthcare workers (HCWs) has been recognized as one of the most critical aspects of high-quality care. Unfortunately, there is still an unmet need for the assessment of compassionate care from the patient’s perspective. During the COVID-19 pandemic, many new rules were enacted to tackle the raging pandemic, which raised concerns about its effect on compassionate care. Methods: A cross-sectional study involving 315 patients from three public hospitals was conducted during the conditional movement control order (CMCO). A self-administered Malay version of the Relational Aspect of Care Questionnaire (RAC-QM) was used to assess compassionate care. Multiple linear regression was used to determine the predictors. Results: More than 90% of the patients were Malays, Muslims, and fell under the B40 household income category. Companions were present for 51.7% of the patients, but 75.2% had no visitors. All hospitals received scores of more than 90%. Occupation (student, *p* = 0.032), dependency level (total dependent, *p* < 0.001), and household income level (M40, *p* = 0.027) were the statistically significant predictors for compassionate care. Conclusions: The current study revealed that compassionate care to patients was not compromised during the pandemic. Patients with disabilities or financial constraints are more likely to experience less compassionate care, while students are generally more satisfied. This study may provide clues for hospital administrators and policymakers regarding the vulnerable group of patients. It also provides opportunities for future research to study the perspective of HCWs.

## 1. Introduction

Compassion and the healthcare system have always been regarded as inseparable entities. The nature of healthcare is to treat and alleviate any suffering that causes or is caused by the disease. Every healthcare worker (HCW) has sworn an oath saying, “I will remember that there is an art to medicine as well as science, and that warmth, sympathy, and understanding may outweigh the surgeon’s knife or the chemist’s drug” [1]. The oath is not only a professional oath that every healthcare worker has to embrace, but also a code of conduct expected by the public. Essentially, it means recognizing the concerns, distress, and suffering of patients and their families and taking action to relieve them. The oath is expressed as affective, cognitive, and behavioral processes [2]. Patients define it as “a virtuous response that seeks to address the suffering and needs of a person through relational understanding and action” [3]. It also embraces patient-centered care’s emotional and psychosocial aspects and the patient’s innate need for human connections and relationships.

The ability to recognize suffering, which flourishes into compassionate behavior, is not limited towards others only; it also allows us to receive compassion from others and from ourselves. According to the event system theory, people will react to an event differently in accordance with their perception of the event’s strength. Event strength can be defined according to its novelty, disruption, and criticality [4]. The stronger an event is, the more motivated individuals become to act and ensure their survival. The COVID-19 pandemic is considered a significantly strong event, since it was a new disease that led to much disruption and put many systems in the world in a critical phase [5,6]. There are many revelations regarding the suffering that HCWs experienced, especially during the peak phase of the pandemic. The conspicuous pandemic has likely ignited compassionate care at work in order to mitigate the effects of the perceived strength of the pandemic on individuals’ suffering. Some might not realize it, but recent studies have illustrated the connections between the pandemic situation and compassionate care [2,7,8,9]. In centers that had enough resources and emphasized the importance of compassion at work, the members of the organization felt cared for and thus experienced less damage [10]. However, unfortunately, due to the intensity of the COVID-19 pandemic, many healthcare providers were not able to adequately prepare their members to face adversity. Many HCWs suffered from secondary traumatic stress and compassionate fatigue, which led to burnout.

Compassionate care has been proven to increase patients’ hopes for recovery, accountability, and control over their health [11,12]. Enforcing compassionate care is beneficial to patients and saves time and costs for the healthcare system [13]. A rapid influx of literature on compassionate care has been observed in the past half-decade. However, most studies focused on healthcare providers’ perceptions rather than patients’. Studies on interpersonal factors related to compassion and its barriers and enablers are still lacking [14]. A study done in Victorian hospitals to analyze the types of complaints revealed that most patients complained about the lack of a proper connection and their experiences of inconsiderate, uncompassionate, and unfriendly relationships with nurses [15]. In a study conducted among medical and surgical inpatients in Pakistan, it was found that less than half of the patients were satisfied with the care provided to them. Ninety percent of the patients were uncomfortable with the nurses’ communication style [16].

In Malaysia, a literature search yielded nil results regarding compassion, which may be attributed to the lack of validated measurement tools to assess compassion [17]. Only recently has the Malay version of the Relational Aspect of Care (RAC-QM) been validated and shown to have good psychometric properties [18]. With an appropriate measurement tool, compassionate care can be validly measured. Associated factors can be studied to allow improvement measures to be taken. Previous studies found several factors that may affect patients’ perceptions of compassionate care, such as ethnicity, level of education, marital status, health status, frequency of service use, income, age, gender, and psychosocial support. However, most of these studies’ findings contradict each other and are inconclusive [19,20,21,22,23,24,25,26,27,28,29,30,31].

Falling ill is one of the most significant tests a person could face, and they could easily fall into a state of vulnerability toward adverse health outcomes, such as disease-associated complications and mental distress. Strong psychosocial support has long been recognized as a positive predictor toward good prognosis. Previous studies have found that those with wide social networks have a decreased risk of mortality compared to those with low psychosocial support [32]. Admission to a ward for any reason can be daunting to patients. Not only are patients wary about their disease, but disruptions to their regular daily routine and non-familiarity with the new environment can also invite unnecessary anxiety. Acquaintances, family members, and friends are essential to easing recovery while patients are in the hospital. The availability of companions or visitors during admission is one form of psychosocial support that can mediate a good patient experience [33,34,35]. A study to evaluate the effect of visitor restrictions on post-operative patients showed that those who had visitor restrictions had the least satisfaction regarding the care received compared to those who had visitors. Patients also rated the psychosocial support received in the form of visitors as high as 84.5% [36]. When the COVID-19 pandemic struck, most hospitals worldwide revised their visitor restriction policies per the World Health Organization recommendation [37]. Various visiting restrictions in different countries and healthcare settings were observed, ranging from absolutely no visitors in any kind of ward to partial visiting restrictions with mitigating procedures and an allowance for stay-in caretakers. Even though the policy was intended for the public interest, it created undesirable repercussions for the patients, their family members, and healthcare providers’ well-being. Patients were reported to experience more pain, reduced ability for self-care, and deteriorating nutritional status, cognitive function, and mental state [38,39,40,41]. Healthcare workers also suffer an increased workload when taking care of patients while, at the same time, regularly updating family members through other channels, such as phone calls and video conferencing [41,42,43]. In addition, hospitals were critically short-staffed, especially in noncritical wards, as most of the staff were deployed to more critical COVID-19 centers [44,45]. The purpose of this study was to investigate whether these depressing conditions had an impact on the compassionate care given to patients, as well as to identify predictors of healthcare workers’ compassionate care as perceived by patients.

## 2. Materials and Methods

### 2.1. Study Setting and Participants

Since the data collection was conducted during the COVID-19 pandemic under the Conditional Movement Control Order (CMCO) in Malaysia, several special considerations had to be taken. The study was conducted in public hospitals located in Kelantan, a northeastern state of Peninsular Malaysia. The state was selected because interstate travel was prohibited during the MCO. There were nine government hospitals in the state. However, three of the hospitals were designated as quarantine and treatment centers for COVID-19 and were thus excluded. Out of the remaining six hospitals, three hospitals that represented the highest proportion of hospital admissions in the state were selected. They consisted of one state hospital (HRPZ (II)) and two district hospitals (HPM and HTA).

The sample size was estimated using a single mean formula to determine the staff’s compassionate care score. At a 95% confidence interval, 20% withdrawal, precision set at three, and a standard deviation of 16.9 based on population satisfaction with care, the required sample size was determined to be 328 patients [46]. To assess the determinants that affected the scores, the sample size was calculated using G* Power software version 3.1 (Heinrich Heine University Düsseldorf, Düsseldorf, Germany). Allowing type 1 error at 0.05 with a statistical power of 80%, medium effect size of 0.15, and 20 expected predictors will require at least 157 samples. With an estimated 20% dropout rate, 188 samples were required. As a result, the largest sample size required to achieve the study’s objectives was 328. Proportionate to the admission rates, 60% of the study participants were selected from HRPZ (II) and 20% from HTA and HPM.

A meeting was conducted with the head nurse to brief them about the study. Also, the standard operating procedure peculiar to conducting research during CMCO was carefully discussed. The study was performed toward the later phase of pandemic, during which Malaysia was about to enter the recovery phase. Ward selection was also made since some wards had restricted entry due to the COVID-19 pandemic. Only nonCOVID wards were selected for the study. The expected daily bed occupancy was noted during the meeting to help determine the sampling size. Stratified proportionate sampling with respect to the number of beds was done to determine the number of participants needed from each ward. The data collection was completed in six weeks. The eligible study population was determined according to the study criteria on the day of data collection. The study participants were selected through random computer-generated numbers based on their bed numbers. The inclusion criteria of the study were those aged 18 and above who had been in the ward for at least 24 h, were able to read and write, and were clinically stable enough to answer the questionnaire by themselves. Those who consented to participate were given the paper-based proforma, which contained RAC-QM and related questions about patients’ backgrounds. They were asked to read the patient information sheet and answer the questions by themselves. Completed questionnaires were collected afterwards by the researcher.

### 2.2. Relational Aspect of Care Questionnaire Malay Version (RAC-QM) 

The original English version of the RAC-Q was developed in 2017 by the Picker Institute of Europe and the University of Oxford. The questionnaire was developed using a combination of quantitative, qualitative, and participatory research approaches [47]. The original questionnaire had 20 items but was successfully reduced to 12 with only one domain. It has an excellent psychometric property with Cronbach’s α = 0.95, McDonald’s Omega coefficient (ω) = 0.05, and factor loadings 0.46–0.87. Comparative Fit Index (CFI) = 0.89, Goodness of Fit Index (GFI) = 0.99, Adjusted Goodness of Fit Index (AGFI) = 0.99, and GFI without diagonal values = 0.99 [47]. It was then translated into Malay and maintained its strong psychometric properties. The standardized factor loading range was from 0.40 to 0.73, with CFI = 0.92, Tucker–Lewis Fit Index (TLI) = 0.90, Root Mean Square Error of Approximation (RMSEA) = 0.06, and Standardized Root Mean Square Residual (SRMR) = 0.07. It has good reliability, with Cronbach’s α = 0.86, a composite ratio of 0.86, and an AVE of 0.34. The questionnaire contained 12 items. Five of the items had three answer options, while another seven questions had four answer options. All except questions number four and number twelve provided answer options in the agreement form, while questions number four and twelve provided answer options in frequency form. For answers in agreement form, four points were given to the “definitely true” answer; three points were given to the “mostly true” answer; the “definitely false” answer was given one point; and those with “unsure” were given two points. For answers in frequency form, four points were given for “every time;” three points were given for “sometimes;” one point was given for “never;” and two points were given for “unsure.” Therefore, the maximum score was 48 points, while the minimum was 12 points. The score is then converted to a percentage. The higher the score, the more compassionate the healthcare is, as perceived by the patients [18].

### 2.3. Statistical Analysis

Data were analyzed using the Statistical Package for the Social Sciences (SPSS) software version 27 (IBM, SPSS Company, Armonk, NY, USA). Preliminary data screening was done for missing values or possible incorrect data entries. Normality was checked using a histogram and a box-and-whisker plot. Numerical data were presented as means (SD) or medians (IQR), while categorical data were presented as frequencies (percent). The scores given by the patients were presented in percentages. Simple linear regression was used to establish the predictors for compassionate care. The analysis was performed for each independent variable, one at a time. The factors that had *p*-values of more than 0.25 and were clinically relevant were selected and analyzed using forward, backward, and stepwise methods in multiple linear regression. Adjusted *b*-coefficients (adjusted to the other variables) were reported. Factors with *p*-values of 0.05 and below were considered significant and included in the model. The two-way interactions between the individual independent variables were checked one by one. The interaction term was created before the analysis. The interaction term was included in the model if the *p*-value was less than 0.05. Model assumptions were checked by examining differences between each response variable’s observed and predicted value (residuals). The assumption of linearity, normality of residuals, and equal variance of residuals were checked by plotting scatter plots.

### 2.4. Ethical Consideration

Ethical clearance for this research was obtained from the Human Research Ethics Committee of Universiti Sains Malaysia (JEPeM Code: USM/JEPeM/21030208) and the National Medical Research Register (NMMR) Malaysia: NMRR-21-344-58027. The confidentiality of the data was strictly prioritized.

## 3. Results

Data from 315 inpatients were eligible for analysis. Sociodemographic, medical, and current admission backgrounds were analyzed and presented descriptively. Simple linear regression and multiple linear regression were conducted to determine the predictors of the compassionate care score.

### 3.1. Sociodemographic Characteristics of Inpatients

Table 1 shows the sociodemographic characteristics of the patients. Malays and Muslims constituted more than 95% of the patients, and almost 90% were classified under the B40 income category based on the Household Income and Basic Amenities survey of the 2019 Department of Statistics Malaysia. In Malaysia, household income can be classified into three categories: B40, M40, and T20. B40 represents the lower income category, which constitutes the bottom 40% of income strata, which, as of 2021, refers to households with incomes of Ringgit Malaysia (RM) 4850 or below. M40 represents the middle-income category, which refers to families with incomes between RM 4851 and RM 10,959. Finally, T20 represents the top 20% of Malaysian household income, with RM 10,960 or more [48]. Approximately 20% of the patients received tertiary education or higher. For occupational status, more than 30% were unemployed.

### 3.2. Medical and Current Admission Background

Patients’ medical backgrounds and details about their current admission were inquired about and presented in Table 2. On average, the patients spent three days in the ward when they took the survey. As expected, the majority (80.6%) were admitted for emergency causes, and more than half (58.1%) had been admitted to the same hospital more than once. More than half of the patients had a caretaker while in the ward, and 70% did not receive visitors during their stay. Looking at the patients’ quality of life, more than half (54.6%) needed at least some help to move; less than half (48.3%) had no problem performing self-care; about 60% needed help in their activities of daily living; and more than 60% were suffering from severe pain or discomfort. About 60% also found that they were unaffected by the no-visitor policy.

The bed occupancy rates of the hospitals are summarized in Table 3. HRPZ (II) had the highest bed occupancy rate (95.5%), followed by HPM and HTA at 67.5% and 58.4%, respectively.

### 3.3. Patients’ Responses to the Questionnaire

Table 4 summarizes the responses from the patients. All 12 questions were in positive forms. In general, more than 70% of the responses were categorized as “definitely true” or “every time,” while 23.4% were categorized as “mostly true” or “sometime.” Only a small proportion of the responses were categorized as “definitely false” or “never,” with a similar proportion for “unsure.” By item, question number one, which asked about whether the staff introduced themselves before treating, received the lowest percentage (41.6%) for the maximum score (4). Almost 10% of patients answered that the staff had never introduced themselves to the patients. The second item that received a lower percentage (48.9%) for maximum score was question number two, which inquired about how much the staff members took the time to get to know the patients as individuals. Among all the items, patients complimented the staff on their confidence the most (86.3%) through item number seven. The item that received the next highest percentage for the maximum score was question number 10; 86.0% of the patients answered that the staff made them feel safe all the time.

### 3.4. Compassionate Care as Perceived by Patients in Public Hospitals in Kelantan

One hundred seventy-nine (56.8%) of the patients had been admitted to the same hospital before, and 60% felt there was no difference in terms of the compassionate care given by the HCWs during the current admission in relation to previous ones. Another 34.4% felt that the HCWs were more compassionate, and only a minority of 5.6% felt that the HCWs were less compassionate compared to past admissions. The average score for HRPZ (II) was 90.65% (12.00), while the district hospitals scored slightly higher, with 91.52% (8.00) and 93.83 (8.05) for HTA and HPM, respectively.

### 3.5. Predictors of Compassionate Care Perceived by Patients 

In an attempt to understand the factors that could affect the compassionate care score as perceived by patients during their stay, all the variables in the study were analyzed using regression analysis. From preliminary data, this current study found that occupation, household income, availability of companions while in the ward, ability for self-care, and ability to move around were statistically significant predictors (*p*-value < 0.05), when adjusted to each other, as summarized in Table 5. Students were found to give scores that were 4.66% higher (0.59, 8.73 95% CI), with a *p*-value of 0.03 compared to other occupations. Patients who were totally dependent on others to move also perceived the HCWs as more compassionate by 7.50% (2.61, 12.39 95% CI). Patients from the middle-income category (M40) gave compassionate scores that were 5.77% lower (−10.10, −1.44 95% CI). Patients who were partially dependent with regard to self-care ablity (SCA) also perceived the HCWs as less compassionate (−7.16, −0.57 95% CI). Patients who were totally dependent on others to perform their self-care had much lower perceptions of compassionate care by 12.35% (−17.88, −6.81 95% CI). Patients who were allowed to have companions while in the ward also perceived HCWs as being less compassionate by 3.13% (−5.85, −0.41 95% CI). About 15.6% of the variance in the score was due to the significant predictors mentioned.

From the preliminary main effect model, two interaction terms were found to be significant: the interaction between being totally dependent for self-care with being in the middle-income category and the interaction between being totally dependent for self-care with being totally dependent for mobility. Therefore, interaction terms were included in the final model. In the final model, students and interaction between being totally dependent for self-care and mobility were positively and significantly associated with compassionate care, with *p*-values of 0.02 and <0.001, respectively, while partial and total dependence for mobility were significantly and negatively associated with compassionate care, with *p*-values of 0.03 and <0.001, respectively. In addition, the interaction term between total dependence for self-care with the total of being in the middle-income category was also significantly and negatively associated with compassionate care, with a *p*-value of 0.006. No multicollinearity was detected, with Variance Inflation Factor (VIF) of the variables ranging from 1.09 to 3.59. Linearity, homogeneity of variance, and normality were tested to ensure that model assumptions were made. Here, 20.7% of the variance was contributed by the predictors. According to the final model, compassionate care as perceived by patients can be predicted using the following equation:

Compassionate care score = 93.71 + (4.93 * student) − (3.65 * Partial dependent for self-care) − (29.57 * Total dependent for self-care) − (20.71 * (Total dependent for self-care × middle-income group)) + (22.15 * (Total dependent for self-care × total dependent for mobility))

Students give scores that are higher by 4.93% compared to other occupational classes. Those who were totally dependent for self-care and mobilization also gave scores that were higher by 22.15% for the compassionate care provided by the HCWs. Contrarily, patients who were partially dependent for SCA gave 3.65% lower scores. Those who were totally dependent for self-care gave even much lower scores, with a reduction of 29.57%. In addition, patients who were totally dependent for self-care and from the middle-income group also gave lower scores by 20.71%. A summary of the findings is shown in Table 6.

## 4. Discussion

This study aimed to reveal patients’ perspectives on compassionate care served by public hospital healthcare workers and the associated predictors.

### 4.1. Compassionate Care during the COVID-19 Pandemic

Healthcare systems worldwide are known to suffer from a scarcity of resources, even before the catastrophic effects of the pandemic. The pandemic added another layer of burden to the overstretched HCWs in the form of a sudden surge in the demand for healthcare. Deployment of HCWs to more critical and COVID-19-related areas that were unfamiliar to them could lead to disorientation and distress. PPE shortages and unsafe working environments contributed to the disproportionate deaths among HCWs. This not only further aggravated the staff shortage, but also caused grief and distress among HCWs. They were subjected to long working hours and had to face suffering patients and family members in high-intensity situations. This has been shown to cause burnout and compassion fatigue among HCWs [49,50,51]. Naturally, HCWs who suffer from burnout will provide low-quality care.

Interestingly, our study revealed that the majority of patients found that HCWs have successfully remained compassionate, and a good proportion even thought that HCWs were even more compassionate now than before the pandemic. Even with the restrictions regarding visitors and the strict requirements for caretakers, most patients claimed that they were not affected by the change in policy. They could also appreciate the compassionate care served by the HCWs. This could be attributed to the reduced number of admissions to the wards during the pandemic. There was a significant reduction in cases unrelated to COVID-19 admitted during the pandemic [52,53,54]. Therefore, in nonCOVID-19 areas, the workload was less than before in terms of admissions. This could have allowed HCWs to provide high-quality care. The study was also conducted during the later phase of the pandemic, wherein the number of COVID-19 cases had significantly decreased. Most of the HCWs who were redeployed to serve in critical areas had been called back. Prolonged suffering fighting through the pandemic may have also activated compassionate care among HCWs, changed their perspectives on life, and echoed the compassionate care to the patients [8,10]. Acts of solidarity and support from the public also kept the motivation strong. In the media, images of heroic HCWs, encouraging words, and free meals from individuals and companies in support of working HCWs were heartwarming [55,56].

Another study that assessed patient experience pre- and post-pandemic in Italy showed that patients had a much better experience during the pandemic. The elements that had higher significance were related to emotional support and communication. They found that their fears and anxieties were addressed more by clinicians. The patients also benefited from good rest while in the ward due to ward silence. The study concluded that the difference in the finding was due to improved awareness of the significant health crisis, which consequently increased patient recognition and understanding of the great difficulties that HCWs faced [57]. Skewness toward higher scores is also commonly seen in many patient-reported experiences. This may be attributed to a number of factors, including signs of appreciation, showings of respect, acts of generosity, or social desirability bias, which could be more prominent in this study’s setting [58].

The analysis of responses to each question could provide guidance regarding aspects of compassionate care that need more improvement. Among the twelve items, HCW’s most lacking attribute was the most basic introduction of oneself to the patients. Even though this was taught during medical and nursing school, it seems insufficiently emphasized among HCWs in Malaysia. This basic art of communication is desired by patients [59,60,61]. However, extensive layers of PPEs were required, and social distancing created substantial obstacles to traditional communication. Facial expressions and emotions became difficult to assess [62]. In addition, the extra protection required during the pandemic was associated with other problems, such as heat stress and dermatological problems, which led to impaired work performance [63,64,65]. Due to these limitations, the duration and frequency of ward rounds were reduced. The less time spent with the patients, the less that relational connections were made. The findings of the current study revealed that more than half of the patients perceived that HCWs did not take the opportunity to get to know them. Some of the patients were also not involved in the decision making related to them. However, most patients complimented the HCWs’ confidence and competency while caring for them and making them feel safe. Other than that, patients seemed satisfied with HCWs’ active learning, and they put the patients at ease by being friendly in conversation.

### 4.2. Predictors for Compassionate Care as Perceived by Patients

For improvement to take place, the predictors that could affect compassionate care must be understood. Patients’ experiences with compassionate care are indeed multifactorial. The compassionate care could be influenced by HCWs, patients, and environmental factors. This study concentrated more on patients’ characteristics and limited aspects of hospital factors. In the final model, after adjusting for other variables, three significant predictors were revealed: occupation, self-care dependence, and having midlevel income. Compared to those who were unemployed, students tended to give a slightly higher score. This factor may be related to their younger age, healthier status, and lower level of education. This finding is supported by other studies that found that those with lower education levels have lower expectations of the system [20,21]. Other than that, patients who were dependent on others for self-care tended to perceive healthcare workers as less compassionate. Understandably, those with some disabilities would require much assistance. Routinely, nurses would help with essential self-care, such as sponging and diaper changing for dependent patients. Other self-care activities are typically performed by caretakers or visitors. During the restriction order, however, visitors had limited access to the ward and were not permitted to be at the patient’s bedside. Unlike other dimensions such as mobility and daily living activity, these activities are typically reduced while admitted to the ward, even among those who typically have no problem with it. Therefore, the patients could not detect any difference. Those who were dependent on others for self-care and came from the middle-income group also tended to give a lower score for compassion. In addition to the disability they have, coming from a middle-income household could put these patients at vulnerability. Individuals in middle-income groups are considered disadvantaged in comparison to those in low-income groups because they are usually not eligible for discounts, rebates, or incentives that are traditionally offered to low-income groups.

This study also determined if hospital factors could affect a patient’s perception of compassionate care. Our study found that the type of hospital and bed occupancy rate were not directly related to perception. This study also investigated whether the visitor restriction policy affected the patients. Univariable analysis showed that the availability of a caretaker at all times while in the ward significantly reduced the perceived compassionate care. However, the result had no significant effect when other variables were adjusted for in the multivariable analysis. Our finding contradicts many other studies that found that visitor restrictions have more negative impacts than positive ones [66]. We are unaware of why some patients still received visitors and why caretakers were allowed. Generally, all public hospitals in Kelantan did not allow it unless in exceptional circumstances, such as psychiatric patients or highly dependent ones.

This study should be considered in light of its limitations. This study was conducted during the later phase of the COVID-19 pandemic. This was done when the cases were plateauing, and some hospitals had eased the visiting and companion restrictions. Therefore, the results may not be representative for all phases of the pandemic. Additionally, the study was conducted in public hospitals and involved nonpaying patients who were mainly unemployed, were not highly educated, and had repeated hospitalizations, which may have altered their expectations of the free service they received. Since ward admission was also highly restricted, this may have led to homogeneity of participants, and the results may not represent the actual population in Malaysia. Kelantan’s population is also largely Malays and Muslim-dominated; the representation of ethnic and religious minorities was not well presented, which limits the study’s generalizability. Furthermore, sampling could only be done in Kelantan, since interstate travel was prohibited. This study was also the first of its kind in Malaysia; therefore, no scores or levels of compassion could be taken as a comparison or benchmark. Due to a lack of data on compassionate care, the researcher decided to use other relational aspects of care keywords, such as empathy, sympathy, patient experience, and satisfaction, as the basis for discussion.

## 5. Conclusions

Compassionate care is a vital aspect of high-quality health services. During the COVID-19 pandemic, it was feared that the provision of compassionate care to patients would be affected by the unfavorable situation. However, this study found that HCWs successfully remained compassionate in their service. Among the elements of compassionate care, HCWs need to improve on their initiative to introduce themselves to patients and learn how to get to know the patient as a person. Patients with disabilities who rely on others for self-care and mobilization are perceived to receive less compassionate care. Furthermore, patients with lower incomes are more likely to receive less compassionate care. Although this study found that the pandemic had no negative impact on compassionate care, future studies need to be conducted during the critical phase of the pandemic and on different groups of patients. Further study is needed from the perspective of HCWs to complement the findings.

## Figures and Tables

**Table 1:** Sociodemographic characteristics of inpatients (*n* = 315).

Characteristics	*n* (%)	Mean (SD)
Age		
Gender		43.8 (17.8)
Male	137 (43.5)	
Female	178 (56.5)	
Race		
Malay	305 (96.8)	
Chinese	7 (2.2)	
Siamese	2 (0.6)	
Others	1 (0.3)	
Religion		
Islam	306 (97.1)	
Buddhism	9 (2.9)	
Marriage Status		
Married	220 (69.8)	
Single	71 (22.5)	
Separated	24 (7.6)	
Education Level		
No formal	19 (6.0)	
Primary	36 (11.4)	
Secondary	189 (60.0)	
Tertiary	66 (21.0)	
Postgraduate	5 (1.6)	
Occupational Status		
Government worker	40 (12.7)	
Private sector	31 (9.8)	
Self-employed	90 (28.6)	
Student	31 (9.8)	
Pensioner	22 (7.0)	
Unemployed	101 (32.1)	
Household Income		
Less than RM 4850	281 (89.2)	
RM 4850–RM 10,959	24 (7.6)	
RM 10,960 and above	10 (3.2)	

**Table 2:** Medical, support, and current admission background (*n* = 315).

Characteristics	*n* (%)	Mean (SD)
Days of Admission		3 (3.0) *
Distance of Hospital from Home (km)		15 (20.0) *
Self-rated Health Status Score		69.1 (21.1)
Chronic Disease		
None	125 (39.7)	
One disease	87 (27.6)	
More than one	103 (32.7)	
Self-Rated Health Status		
Satisfactory	215 (68.3)	
Not satisfactory	100 (31.7)	
Companion in Ward		
All the time	163 (51.7)	
Occasionally	36 (11.4)	
None	116 (36.8)	
Visitors while in Ward		
Everyday	27 (8.6)	
Occasionally	51 (16.2)	
None	237 (75.2)	
Number of Hospitalizations		
First hospitalization	132 (41.9)	
Repeated hospitalization	183 (58.1)	
Reason for Admission		
Emergency	254 (80.6)	
Elective	61 (19.4)	
Ability to Move		
No problem	143 (45.4)	
Some problem	110 (34.9)	
Totally dependent	62 (19.7)	
Ability for Self-Care		
No problem	152 (48.3)	
Some problem	123 (39.0)	
Totally dependent	40 (12.7)	
The Activity of Daily Living		
No problem	127 (40.3)	
Some problem	130 (41.3)	
Totally dependent	58 (18.4)	
Pain or Discomfort		
None	120 (38.1)	
Some	172 (54.6)	
Very Much	23 (7.3)	
Anxiety or Depression		
None	199 (63.2)	
Some	95 (30.2)	
Very much	21 (6.7)	
Affected by the Hospital Policy		
Yes	130 (41.3)	
No	185 (58.7)	

* median (IQR).

**Table 3:** Bed occupancy rates of the hospitals.

Hospital	Bed Occupancy Rate (%)
State Hospital	
HRPZ (II)	95.5
District Hospitals	
HTA	58.4
HPM	67.5

**Table 4:** Responses to each item in the questionnaire (*n* = 315).

Statements	Responses *n* (%)				Min, Max
	(1)	(2)	(3)	(4)	
1.Adakah staf kesihatan memperkenalkan diri sebelum merawat atau menjaga anda? *Have staff introduced themselves before treating or caring for you?*	28 (8.9)	NA	156 (49.5)	131 (41.6)	1, 4
2. Adakah staf kesihatan mengambil peluang untuk mengenali anda? *Have staff taken the opportunity to learn about you as a person?*	18 (5.7)	21 (6.7)	122 (38.7)	154 (48.9)	1, 4
3.Adakah staff mebuatkan anda berasa selesa dengan bersikap ramah dan mesra semasa berhubung dengan anda? *Have the staff made you feel at ease by being friendly and warm in conversations?*	3 (0.9)	NA	57 (18.1)	255 (81.0)	1, 4
4. Pernahkah staf menunjukkan rasa mengambil berat dan penyayang? *Have staff shown you care and compassion?*	2 (0.6)	3 (0.9)	82 (26.0)	228 (72.5)	1, 4
5. Adakah staf mendengar apa yang anda perkatakan? *Have staff listened to what you have to say?*	4 (1.3)	NA	56 (17.7)	255 (81.0)	1, 4
6.Sepanjang anda berada di hospital, adakah anda mendapat interaksi yang secukupnya dengan staf? *During your time in the hospital, have you had enough contact with the staff?*	3 (0.9)	NA	70 (22.2)	242 (76.8)	1, 4
7. Adakah staf kelihatan yakin dan dapat melaksanakan tugas mereka Ketika merawat anda? *Do staff appear confident and able to perform their tasks when caring for you?*	0 (0)	3 (1.0)	40 (12.7)	272 (86.3)	2, 4
8. Adakah anda mempunyai cukup masa untuk membincangkan masalah kesihatan atau perubatan anda dengan doktor ataupun jururawat? *Have you had enough time to discuss your health or medical problems with a doctor or nurse?*	3 (1.0)	1 (0.3)	81 (25.7)	229 (72.7)	1, 4
9. Adakah anda telah dilibatkan Bersama sepertimana yang anda mahu didalam menentukan penjagaan dan rawatan anda? *Have you been involved as much as you want to be in decisions about your care and treatment?*	6 (1.9)	11 (3.5)	69 (21.9)	229 (72.7)	1, 4
10. Sepanjang anda berada di hospital, adakah staf membuat anda berasa selamat? *During your time in the hospital, have staff made you feel safe?*	2 (0.6)	2 (0.6)	39 (12.4)	272 (86.)	1, 4
11. Pernahkah anda menerima sokongan seperti yang anda perlukan daripada staf? *Have you received as much support as you have needed from the staff?*	2 (0.6)	4 (1.3)	70 (22.2)	239 (75.9)	1, 4
12. Secara keseluruhan, adakah anda rasa anda telah dilayan dengan hormat dan bermaruah sepanjang anda berada di hospital? *Overall, do you feel you have been treated with respect and dignity while in the hospital?*	0.00	NA	47 (14.9)	268 (85.1)	3, 4
Total	71 (1.8)	35 (1.5)	889 (23.4)	2774 (73.3)	

(1) Definitely false or never; (2) unsure; (3) mostly true or sometime; (4) definitely true or every time. NA = the score option is not applicable for the question.

**Table 5:** Preliminary main effect model of compassionate care perceived by patients (*n* = 315).

Predictors	Simple Linear Regression			Multiple Linear Regression		
	Crude b ^a^ (95%CI)	*p*-Value	R2	Adjusted b ^b^ (95% CI)	T-Stat	*p*-Value
Occupation		0.05	0.04			
Unemployed	0					
Student	4.72 (0.42, 9.01)	0.03		4.66 (0.59, 8.73)	2.251	0.03
Government	2.57 (−1.34, 6.49)	0.20		3.11 (−0.84, 7.05)	1.551	0.12
Private sector	0.55 (−3.75, 4.85)	0.80		1.31 (−2.82, 5.44)	0.625	0.53
Self-employed	0.38 (−2.66, 3.42)	0.81		0.79 (−2.19, 3.78)	0.522	0.60
Pensioner	−4.36 (−9.23, 0.56)	0.08		−3.49 (−8.15, 1.18)	−1.469	0.14
Household Income		0.06	0.02			
B40	0					
M40	−5.05 (−9.52, −0.59)	0.03		−5.77 (−10.10, −1.44)	−2.624	0.01
T20	2.35 (−4.75, 9.46)	0.52		−0.87 (−7.71, 5.98)	−0.249	0.80
Companion in Ward		0.03	0.04			
None	0					
Sometime	−0.71 (−4.68, 3.25)	0.72		−1.16 (−4.96, 2.63)	−0.604	0.54
All the time	−4.23 (−6.76, −1.71)	0.001		−3.13 (−5.85, −0.41)	−2.267	0.02
Self-Care Ability (SCA)		<0.001	0.07			
Independent	0					
Partial	−3.70 (−6.14, −1.27)	0.003		−3.86 (−7.16, −0.57)	−2.309	0.02
Totally dependent	−7.90 (−11.49, −4.31)	<0.001		−12.35 (−17.88, −6.81)	−4.387	<0.001
Mobility		0.03	0.02			
Independent	0					
Partial	−3.17 (−5.77, −0.57)	0.02		0.86 (−2.31, 4.04)	0.535	0.59
Totally dependent	−3.13 (−6.27, 0.02)	0.05		7.50 (2.62, 12.39)	3.022	0.04

^a^ Crude regression coefficient ^b^ Adjusted regression coefficient R^2^ = 15.6%. The model fits reasonably well. Model assumptions are met, and no multicollinearity problem is present.

**Table 6:** Predictors of compassionate care score perceived by patients (*n* = 315).

Predictors	Simple Linear Regression			Multiple Linear Regression		
	Crude b ^a^ (95%CI)	*p*-Value	R2	Adjusted b ^b^ (95% CI)	*t*-Test	*p*-Value
Occupation			0.04			
Unemployed	0					
Student	4.72 (0.42, 9.01)	0.032		4.93 (0.97, 8.90)	2.45	0.02
Government	2.57 (−1.34, 6.49)	0.197		3.06 (−0.78, 6.90)	1.57	0.19
Private sector	0.55 (−3.75, 4.85)	0.802		1.88 (−2.15, 5.91)	0.92	0.36
Self-employed	0.38 (−2.66, 3.42)	0.806		1.38 (−1.54, 4.30)	0.93	0.35
Pensioner	−4.36 (−9.29, 0.56)	0.082		−1.71 (−6.33, 2.90)	−0.073	0.47
Household Income			0.02			
B40	0					
M40	−5.05 (−9.52, −0.59)	0.027		−4.09 (−8.51, 0.32)	−1.82	0.07
T20	2.35 (−4.75, 9.46)	0.515		−0.52 (−7.18, 6.14)	−0.16	0.88
Companion in ward			0.04			
None	0					
Sometime	−0.71 (−4.68, 3.25)	0.724		−1.17 (−4.86, 2.51)	−0.63	0.53
All the time	−4.23 (−6.76, −1.71)	0.001		−2.51 (−6.17, 0.15)	−1.86	0.06
Self-Care Ability (SCA)			0.07			
Independent	0					
Partial	−3.71 (−6.14, −1.27)	0.003		−3.65 (−6.86, −0.43)	−2.23	0.03
Totally dependent	−7.90 (−11.49, −4.31)	<0.001		−29.57 (−40.91, −18.22)	−5.13	<0.001
Mobility			0.02			
Independent	0					
Partial	−3.17 (−5.77, −0.57)	0.051		0.53 (−2.56, 3.62)	0.34	0.74
Totally dependent	−3.13 (−6.27, 0.02)	0.017		4.72 (−0.30, 9.73)	1.85	0.07
*SCA_M40	−28.17 (−42.84, −13.52)	<0.001	0.04	−20.71 (−35.32, −6.09)	−2.79	0.01
#SCA_Mobil	−3.96 (−7.60, −0.32)	0.033	0.02	22.15 (9.85, 34.46)	3.54	<0.001

^a^ Crude regression coefficient; ^b^ Adjusted regression coefficient R^2^ = 20.7%; #SCA_MOBIL: Interaction term between total dependence for mobility with total dependence for self-care; *SCA_M40: Interaction term between total dependence for self-care with being in the M40 income category.

## Data Availability

The data are not publicly available due to privacy and confidentiality. However, restrictions applied to the availability of hospital data, which are available from the authors with the organization’s permission.
